# Refusal of Surgery in Pituitary Adenoma Patients: A Population-Based Analysis

**DOI:** 10.3390/cancers14215348

**Published:** 2022-10-30

**Authors:** Jack L. Birkenbeuel, Brandon M. Lehrich, Khodayar Goshtasbi, Arash Abiri, Frank P. K. Hsu, Edward C. Kuan

**Affiliations:** 1 Department of Otolaryngology–Head and Neck Surgery, University of California, Irvine, CA 92868, USA; 2 Medical Scientist Training Program, School of Medicine, University of Pittsburgh, Pittsburgh, PA 15260, USA; 3 Department of Neurosurgery, University of California, Irvine, CA 92868, USA

**Keywords:** pituitary adenoma, surgery, surgery refusal, socioeconomic, survival

## Abstract

**Simple Summary:**

This study presents an evaluation of the clinical and sociodemographic factors predictive of surgery refusal in pituitary adenoma (PA) patients and the effect of surgical receipt on overall survival outcomes based on patient data from the National Cancer Database. To our knowledge, the impact of pituitary refusal on outcomes has not been investigated by a research team. Here, we identified age > 65, African American race, increased comorbidities, and government insurance or being uninsured as independent predictors of surgery refusal. We also demonstrate a significant decrease in overall survival in PA patients with macroadenoma who refuse surgery compared to those who receive surgery. We hope these findings can help physicians understand the sociodemographic factors that influence surgery refusal in PA patients, as well as the importance of surgery in appropriate patients with macroadenomas.

**Abstract:**

We characterized the clinical and sociodemographic factors predictive of surgery refusal in pituitary adenoma (PA) patients. We queried the National Cancer Database (NCDB) to identify adult PA patients treated from 2004–2015 receiving or refusing surgery. Multivariate logistic regression and Cox proportional-hazards analysis identified clinical and/or sociodemographic factors predictive of surgery refusal or mortality, respectively. Of the 34,226 patients identified, 280 (0.8%) refused surgery. On multivariate logistic regression, age > 65 (OR: 2.64; *p* < 0.001), African American race (OR: 1.70; *p* < 0.001), Charlson-Deyo Comorbidity (C/D) Index > 2 (OR: 1.52; *p* = 0.047), and government insurance (OR: 2.03; *p* < 0.001) or being uninsured (OR: 2.16; *p* = 0.03) were all significantly associated with surgery refusal. On multivariate cox-proportional hazard analysis, age > 65 (HR: 2.66; *p* < 0.001), tumor size > 2 cm (HR: 1.30; *p* < 0.001), C/D index > 1 (HR: 1.53; *p* < 0.001), having government insurance (HR: 1.66; *p* < 0.001) or being uninsured (HR: 1.67; *p* < 0.001), and surgery refusal (HR: 2.28; *p* < 0.001) were all significant predictors of increased mortality. Macroadenoma patients receiving surgery had a significant increase in overall survival (OS) compared to those who refused surgery (*p* < 0.001). There are significant sociodemographic factors that influence surgery refusal in PA patients. An individualized approach is warranted that considers functional status, clinical presentations, and patient choice.

## 1. Introduction

While the World Health Organization (WHO) now classifies pituitary adenomas (PA) through their adenohypophyseal cell lineage, they have been traditionally categorized and treated based on size (e.g., microadenoma < 1 cm and macroadenoma ≥ 1 cm) and functional status (i.e., functional versus non-functional) [1,2]. The estimated prevalence of PAs are around 17% in the general population based on autopsy and radiologic studies, with many remaining asymptomatic throughout one’s lifetime [1]. Nonfunctioning PA patients may be managed through observation or medical management; however, those causing symptoms (e.g., mass effect, vision loss, endocrinopathy) and/or prolactinomas that fail to respond to medical therapy warrant surgical intervention [3,4,5].

Surgery for PA, including endoscopic, microscopic, and open approaches [6], generally produces favorable outcomes, including improvement in quality of life, improvement in vision in up to 89% of patients, and biochemical remission rates ranging from 42–100% based on size and type of secreting PA (e.g., Cushing disease, acromegaly, prolactinoma) [7,8,9]. While there are inherent surgical risks, including post-operative CSF leak, meningitis, hypopituitarism, diabetes insipidus [10], mortality and major complication rates are very low at <0.5% and 1–3%, respectively. However, not all PA surgical candidates elect to undergo surgery. The role of surgery refusal on patient clinical outcomes has been previously reported in the setting of colon, breast, thyroid, head and neck squamous cell carcinoma (HNSCC), and laryngeal cancers [11,12,13,14,15] While these studies have reported poor outcomes in eligible patients electing for surgery refusal [11,12,15,16] to our knowledge, there are no reports on clinical outcomes in PA patients refusing surgery. Therefore, this study aims to characterize the factors associated with and clinical outcomes of surgery refusal in PA patients.

## 2. Materials and Methods

This work was exempt from Institutional review board (IRB) approval given lack of personal health identifiers in the National Cancer Database (NCDB, https://www.facs.org/quality-programs/cancer-programs/national-cancer-database/, accessed on 5 June 2021). The NCDB is a publicly accessible, comprehensive cancer registry in the United States (US) which provides a large sample of cancer patients with presenting clinical and sociodemographic information in the US [17]. All patients between 2004 and 2015, with a diagnosis of PA through International Classification of Disease (ICD) codes (C751.0) and histology/behavior codes (8140/0, 8202/0, 8260/0, 8270/0, 8271/0, 8272/0, 8280/0, 8281/0, 8290/0, 8300/0) met initial inclusion criteria. The “REASON_FOR_NO_SURGERY” variable included in the NCDB allowed for the separation of data into two separate cohorts (i.e., surgery refusal and surgery receipt). All participants who refused surgery were eligible surgical candidates and our definition of surgery refusal has been reported in prior NCDB investigations [11,12,13,14,15]. Exclusion criteria included patients <18 years of age, those with pituitary carcinomas, >1 primary non-malignant tumor, palliative care management, treatment at a site other than the NCDB reporting facility, treatments other than for their primary tumor, and unspecified follow-up.

For both treatment cohorts, information on the following clinical and sociodemographic characteristics were provided by NCDB: age, sex, race, tumor size, year of diagnosis, tumor size, Charlson-Deyo Comorbidity (C/D) Index score, treatment facility type or geographic region, insurance type, income quartile, zip code with the percentage of residents receiving a high school diploma (HSD), urban/rural population size, and distance from patient residence to provider (in miles). Tumor sizes < 3 mm or >105 mm were excluded to account for potential reporting errors. Microadenomas were defined as tumors < 1 cm, and macroadenomas as tumors ≥ 1 cm.

The data collected from each patient were analyzed through the statistical programming language R (version 4.0.2; The R Foundation for Statistical Computing, Vienna, Austria) and RStudio (version 1.2.1335; RStudio, Boston, MA, USA). Independent-samples *t*-test and chi-squared test were used to compare differences in baseline continuous and categorical covariates, respectively. Covariates significantly different (*p* < 0.05) on univariate analyses, or those deemed clinically relevant, were included for, and adjusted in, multivariate analyses. Multivariate logistic regression and Cox proportional-hazards analysis identified clinical and/or sociodemographic factors predictive of surgery refusal or mortality, respectively. Kaplan–Meier log-rank test determined significant differences in overall survival (OS) time between PA patients receiving or refusing surgery. This study utilized an α = 0.05 as the threshold for statistical significance.

## 3. Results

Of the 34,226 patients with pituitary adenoma, 280 (0.8%) refused surgery. The mean age (65 ± 17.3) of patients who refused surgery was significantly higher than the mean age (53 ± 15.3) of patients who received surgery (*p* < 0.001) (Table 1). There were significant differences in race, tumor size, C/D Index, facility type, geographic region, insurance status, income level, zip code for the number of residents without HSD, urban or rural population, and distance from patient to provider between those who refused or received surgery (*p* < 0.05) (Table 1).

On multivariate logistic regression, age > 65 (OR: 2.64; *p* < 0.001), African American race (OR: 1.70; *p* < 0.001), C/D index > 2 (OR: 1.52; *p* = 0.047), and government insurance (OR: 2.03; *p* < 0.001) or being uninsured (OR: 2.16; *p* = 0.03) were all significantly associated with surgery refusal (Table 2). Tumor size > 2 cm (OR: 0.70; *p* = 0.01), care at an academic facility (OR: 0.72; *p* = 0.02), or in the west region (OR: 0.59; *p* = 0.02), and increased distance from patient to provider (OR: 0.99; *p* < 0.001) were all significantly associated with surgery receipt (Table 2).

On multivariate cox-proportional hazard analysis, age >65 (HR: 2.66; *p* < 0.001), tumor size > 2 cm (HR: 1.30; *p* < 0.001), C/D index >1 (HR: 2.35; *p* < 0.001), having government insurance (HR: 1.66; *p* < 0.001) or being uninsured (HR: 1.67; *p* < 0.001), and surgery refusal (HR:2.28; *p* < 0.001) were all significant predictors of increased mortality (Table 3). On multivariate cox-proportional hazard analysis, female sex (HR: 0.85; *p* < 0.001), race other than white/African American (HR: 0.79; *p* = 0.02), care at an academic facility (HR: 0.84; *p* < 0.001), income > $48,000 (HR: 0.82; *p* < 0.001), population > 250,000 people (HR: 0.85; *p* < 0.001), and decreased distance from patient to provider (HR: 0.99; *p* = 0.03) were all significant predictors of improved mortality (Table 3).

**Figure 1:**
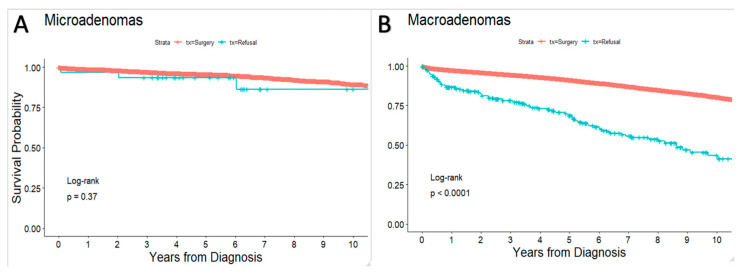
Kaplan–Meier curve of Overall Survival for Pituitary Adenoma Patients Matched on Age, C/D, and Tumor Size comparing Surgery Refusal and Surgery Receipt for (**A**) Microadenomas and (**B**) Macroadenomas.

OS rates at 1, 2, 5 and 10 years in patients who received surgery were 98%, 96%, 92%, and 81%, respectively, whereas OS rates in those who refused surgery were 88%, 84%, 72%, and 48%, respectively (*p* < 0.001). In patients with microadenomas, there were no significant differences in OS between surgery receipt and surgery refusal across all age cohorts (*p* = 0.37) (Figure 1A). In patients with macroadenomas, those who received surgery had significantly improved OS compared to those who refused surgery across all age groups (*p* < 0.001) (Figure 1B).

## 4. Discussion

This is the first study to investigate clinical and sociodemographic factor predictors of surgery refusal, along with its impact on clinical outcomes in PA patients. Our results demonstrated that age > 65, African American race, C/D index > 2, having government insurance or being uninsured were significantly associated with surgery refusal, while tumor size, treatment location, and distance from patient to provider were significantly associated with surgery receipt. Our results also demonstrated significantly worse OS outcomes in those who refused surgery compared to those who received surgery, although this may have been affected by selection bias (e.g., the difference in the risk profile that was presented to the patients). On subgroup analysis, we observed no significant OS differences in PA patients with microadenomas, while we observed significant improvements in OS for PA patients with macroadenomas who received surgery compared to those who refused. Although these results can potentially play a role in discussing PA management options, the authors still advocate for a multifaceted, individualized, and shared decision-making process.

Many sociodemographic factors have been previously reported as predictors of surgery refusal in patients with various cancers. For example, advanced age as a predictor of surgery refusal has been well documented in various malignant tumors, including breast cancer, esophageal cancer, colon cancer, HSNCC, and oral cavity cancer [11,12,14,16,18]. African American race is another sociodemographic factor associated with increased risk of surgery refusal in prostate cancer, colon cancer, HNSCC, laryngeal cancer, and hepatocellular carcinoma [12,14,15,19,20]. Higher comorbidity index, while not reported as frequently, has also been recently associated with surgery refusal in patients with HNSCC. The impact of sociodemographic factors on surgery refusal in patients with anterior skull base tumor has not been well reported previously. While pituitary adenomas are benign tumors with more indolent courses than malignant tumors, they appear to share analogous sociodemographic factors with various malignancies predictive of surgery refusal. Whether this is a true disparity, as in the case of treatment patterns for other tumors, is uncertain, but is worthwhile noting in this highly prevalent disease.

One other important sociodemographic factor associated with surgery refusal in this study is having government insurance or being uninsured. The presence of government insurance has also been associated with surgery refusal in individuals with HNSCC, prostate, esophageal, colon, and oral cancers [12,16,18,19,21]. Furthermore, lack of insurance has been reported as a factor for surgery refusal in HNSCC, esophageal, colon, and oral cancers [12,16,18,21]. While these clinical and sociodemographic factors have not been previously reported for pituitary adenomas or other anterior skull base tumors, it is important to recognize the influence of these factors on surgery receipt. These findings should alert physicians to contributing factors surrounding surgery refusal to better address and respond to these healthcare disparities.

Our results also demonstrated many factors not associated with surgery refusal, but in fact surgery receipt, including tumor size, treatment location, and distance from patient to provider. Regarding tumor size, previous reports of individuals with HNSCC, oral cavity, and locally advance laryngeal cancers have documented increased association of surgery refusal with increased tumor size [15,18,21]. While surgery refusal with advanced tumor size in these malignancies may be related to them being malignant and therefore inoperable, it is important to acknowledge that our results are different from these prior reports, particularly with benign lesions. We noticed the opposite trend, with larger tumors necessitating surgery receipt. Regarding distance from patient to provider, our findings are in agreement with a prior report on stage I–III rectal cancer, which reported increased surgery receipt in individuals who lived further from their provider’s location [22]. Our findings also are in agreement with a previous study on esophageal cancer that reported increased odds of surgery receipt with increased patient to provider distance [16]. Additionally, our findings revealed that surgery receipt was associated with receiving care at an academic facility, which is consistent with prior studies in individuals with esophageal and oral cavity cancer [16,18]. This finding may provide direction to primary care physicians seeking an appropriate surgeon for patients with pituitary adenomas who require surgery. Interestingly, our report demonstrates increased odds of surgery receipt in the West region compared to Central and East regions of the US. To our knowledge, there are no prior reports with similar findings in other pathologies.

Across all ages studied, our results demonstrated no significant differences in OS between PA patients with microadenomas who received surgery and those who refused. While patients with microadenomas require surgery less frequently than those with macroadenomas, the current standard of care for both non-functioning microadenomas and symptomatic functioning microadenomas, excluding prolactinomas, is surgical resection [23,24,25,26]. Our findings offer new data that OS in patients with microadenomas may not change if patients refused surgery, which can play a role in the shared decision-making process. It should however be noted that this study’s data does not provide information on difference in quality of life (QOL) (which has played a role in prostate cancer [27]), symptoms between cohorts, and disease-specific survival rates. This information is also important to consider, as it has been previously reported that both surgery and conservative management can lead to vision and endocrine recovery in patients with pituitary apoplexy [26]. In contrast, our findings revealed that surgery receipt in patients with pituitary macroadenomas, across all ages examined, conferred improved OS outcomes compared to surgery refusal. These findings are consistent with the standard of care for macroadenomas [3,5,23,24,25]. Careful discussion of treatment options (including surgery, when appropriate) in patients with pituitary macroadenomas continues to be warranted. However, it is also important to keep in mind that PA is a benign tumor, with clinical behavior dissimilar to malignancies, yet may incur risk over time if left untreated (e.g., apoplexy, progressive endocrinopathy). In addition to survival outcomes, surgery receipt in PA patients significantly improves QOL according to a 36-item short form instrument assessing QOL after transnasal, endoscopic pituitary surgery [28].

The findings elicited in this manuscript also highlight the disparities in present US healthcare. The notion that race and socioeconomic status affect individual health decisions and associated health outcomes has been previously well documented [29,30,31,32,33]. These factors affect diseases managed both medically and surgically. For instance, the CDC’s second ‘Health Disparities & Inequalities Report’ reported differences in medically diagnosed diabetes among different races, educational attainment levels, and income ratios [34]. Additionally, a review by Marlow et al. found individuals without insurance or insurance through lower income agencies (e.g., Medicaid) to be less likely to obtain necessary cancer screenings, to present at more significantly advanced stages of disease, and to have worse OS outcomes [29]. As mentioned previously, these socioeconomic factors also affect both the decision to receive surgery and OS outcomes in patients with tumors requiring surgery. Our study confirms these findings for PA patients. Specifically, our findings raise concern that socioeconomic factors may drive decisions on surgery refusal or receipt for PA more than previously expected. For this reason, it is important for physicians to consider these factors to ensure optimal care management irrespective of sociodemographic background to PA patients.

This study has several main limitations, of which some are inherent to NCDB studies. For example, the NCDB data on mortality outcomes does not provide disease-specific survival rates [17]. Instead, they only present all-cause mortality rates, making it difficult to extrapolate PA surgery refusal on disease-specific survival outcomes (i.e., survival may affected by other causes). Second, this study’s cohort may be affected by selection bias, which may reduce the generalizability of the comparisons to the general public. Furthermore, the NCDB does not provide other important clinical variables/outcomes, including changes in symptoms (e.g., headache, vision loss), QOL over time, and indication for surgery. Lastly, the NCDB does not provide information on the functional status of pituitary adenomas. Pituitary apoplexy and secretory tumors tend to necessitate early surgical intervention, whereas for prolactinomas, medical therapy (e.g., cabergoline) tends to be first-line. As such, we were unable to determine the impact of functional status on surgery receipt and outcomes. However, it is of still importance to understand sociodemographic drivers of surgery refusal for PA, and the NCDB is suited as a great database to identify these drivers. While these limitations are important to acknowledge, our findings represent the first report of the clinical and sociodemographic factors associated with surgery refusal, as well as its potential impact on clinical outcomes.

## 5. Conclusions

This is the first study to analyze the role of surgery refusal in PA patients. We demonstrated important clinical and sociodemographic factors, including age >65, African American race, C/D Index > 2, and government insurance or being uninsured are associated with surgery refusal. Additionally, on multivariate analysis, we identified surgery refusal to be predictive of increased mortality, although this may be influenced by selection bias. It is important for physicians to understand the health disparities that influence surgery refusal in these individuals. These results may improve physician awareness on the impact of surgery refusal in patients with PA and other anterior skull base tumors. Individualized treatment paradigms are warranted based on tumor pathology, physician judgement, and patient preferences. Future studies should investigate the role of surgery refusal in subgroups of pituitary adenomas, including functioning and non-functioning adenomas.

## Figures and Tables

**Table 1:** Clinical and sociodemographic factors for pituitary adenoma stratified by surgical refusal or surgical receipt.

Covariate	Surgery Refusal, *n* = 280	Surgery Receipt, *n* = 33,946	*p*-Value
Age, no. (%)			<0.001 *
<65 years	120 (42.9)	25,377 (74.8)	
≥65 years	160 (57.1)	8569 (25.2)	
Sex, no. (%)			0.19
Male	137 (48.9)	17,992 (53.0)	
Female	143 (51.1)	15,954 (47.0)	
Race, no. (%)			<0.001 *
Caucasian	169 (61.7)	25,129 (75.4)	
African American	89 (32.5)	6254 (18.8)	
Other	16 (5.8)	1922 (5.8)	
Year of Diagnosis			0.06
2004–2009	129 (46.1)	13,733 (40.5)	
2010–2015	151 (53.9)	20,213 (59.5)	
Tumor size in mm, mean ± SD	22.7 ± 11.1	24.2 ± 12.0	0.02 *
C/D Index, no. (%)			<0.001 *
0	197 (70.4)	25,721 (75.8)	
1	54 (19.3)	6544 (19.3)	
≥2	29 (10.4)	1681 (4.9)	
Facility Type, no. (%)			<0.001 *
Nonacademic	129 (46.1)	10,683 (31.5)	
Academic	151 (53.9)	23,263 (68.5)	
Geographic Region, no. (%)			0.04 *
Central	108 (42.9)	10,785 (40.2)	
East	111 (44.0)	10,858 (40.5)	
West	33 (13.1)	5188 (19.3)	
Insurance Status, no. (%)			<0.001 *
Private	79 (29.15)	20,159 (60.2)	
Government	179 (66.05)	11,670 (34.9)	
Uninsured	13 (4.8)	1631 (4.9)	
Income, no. (%)			0.01 *
<$48,000	134 (48.4)	13,809 (40.8)	
≥$48,000	143 (51.6)	20,018 (59.2)	
ZIP code, residents w/o HSD, no. (%)			0.004 *
<13%	127 (45.7)	18,447 (54.5)	
≥13%	151 (54.3)	15,397 (45.5)	
Urban/rural population, no. (%)			0.02 *
<250,000 people	50 (18.2)	8173 (24.7)	
≥250,000 people	224 (81.8)	24,899 (75.3)	
Distance from patient to provider in miles, mean ± SD	30.0 ± 58.8	53.5 ± 149.8	<0.001 *

Percentages reflect known or reported values. *p*-value represents *t*-test for continuous and chi-square for categorical variables. C/D Index = Charlson-Deyo Comorbidity Index. * Significant *p*-value.

**Table 2:** Multivariate logistic regression analysis of clinical and sociodemographic factors predictive of surgery refusal (*n* = 280) versus surgery receipt (*n* = 33,946) for pituitary adenoma.

Covariate	OR (95% CI)	*p*-Value
Age		
<65 years	1 ^#^	
≥65 years	2.64 (1.88–3.75)	<0.001 *
Race		
Caucasian	1 ^#^	
African American	1.70 (1.25–2.30)	<0.001 *
Other	1.50 (0.82–2.55)	0.16
Tumor size		
<2 cm	1 ^#^	
≥2 cm	0.70 (0.54–0.93)	0.01 *
C/D Index		
0	1 ^#^	
1	0.83 (0.59–1.15)	0.28
≥2	1.52 (0.99–2.26)	0.047 *
Facility Type		
Nonacademic	1 ^#^	
Academic	0.72 (0.55–0.95)	0.02 *
Geographic Region		
Central	1 ^#^	
East	0.94 (0.71–1.24)	0.66
West	0.59 (0.37–0.91)	0.02 *
Insurance Status		
Private	1 ^#^	
Government	2.03 (1.40–2.96)	<0.001 *
Uninsured	2.16 (1.03–4.08)	0.03 *
Income, no. (%)		
<$48,000	1 ^#^	
≥$48,000	0.89 (0.64–1.24)	0.50
ZIP code, residents w/o HSD, no. (%)		
≥13%	1 ^#^	
<13%	0.88 (0.64–1.21)	0.44
Urban/rural population		
<250,000 people	1 ^#^	
≥250,000 people	1.22 (0.82–1.83)	0.33
Distance from patient to provider in miles, continuous	0.99 (0.98–0.99)	<0.001 *

OR = odds ratio. 95% CI = 95% confidence interval. C/D Index = Charlson-Deyo Comorbidity Index. * Significant *p*-value. ^#^ 1 is the reference value.

**Table 3:** Multivariate cox proportional-hazard analysis of clinical and sociodemographic factors of PA OS.

Covariate	Univariate Analysis		Multivariate Analysis	
	HR (95% CI)	*p*-Value	HR (95% CI)	*p*-Value
Age, y				
<65	1 ^#^		1 ^#^	
≥65	4.89 (4.56–5.24)	<0.001 *	2.66 (2.41–2.94)	<0.001 *
Sex				
Male	1 ^#^		1 ^#^	
Female	0.74 (0.69–0.80)	<0.001 *	0.85 (0.79–0.91)	<0.001 *
Race				
White	1 ^#^		1 ^#^	
African American	1.18 (1.09–1.29)	<0.001 *	1.04 (0.95–1.15)	0.54
Other	0.66 (0.55–0.80)	<0.001 *	0.79 (0.65–0.97)	0.02 *
Tumor Size				
<2 cm	1 ^#^		1 ^#^	
≥2 cm	1.65 (1.53–1.79)	<0.001 *	1.30 (1.19–1.41)	<0.001 *
C/D Index				
0	1 ^#^		1 ^#^	
1	1.92 (1.78–2.08)	<0.001 *	1.53 (1.41–1.67)	<0.001 *
≥2	3.66 (3.29–4.07)	<0.001 *	2.35 (2.10–2.63)	<0.001 *
Facility Type				
Nonacademic	1 ^#^		1 ^#^	
Academic	0.56 (0.52–0.60)	<0.001 *	0.84 (0.78–0.91)	<0.001 *
Geographic Region				
Central	1 ^#^		1 ^#^	
East	0.94 (0.87–1.02)	0.14	1.01 (0.93–1.10)	0.80
West	0.84 (0.76–0.93)	<0.001 *	0.99 (0.89–1.10)	0.87
Insurance Status				
Private	1 ^#^		1 ^#^	
Government	4.17 (3.87–4.50)	<0.001 *	1.66 (1.49–1.84)	<0.001 *
Uninsured	1.79 (1.49–2.15)	<0.001 *	1.67 (1.35–2.06)	<0.001 *
Income				
<$48,000	1 ^#^		1 ^#^	
≥$48,000	0.66 (0.61–0.70)	<0.001 *	0.82 (0.75–0.90)	<0.001 *
ZIP code, residents w/o HSD				
≥13%	1 ^#^		1 ^#^	
<13%	0.75 (0.71–0.81)	<0.001 *	0.95 (0.87–1.04)	0.26
Urban/rural population				
<250,000 people	1 ^#^		1 ^#^	
≥250,000 people	0.72 (0.67–0.78)	<0.001 *	0.85 (0.78–0.93)	<0.001 *
Distance from patient to provider, continuous	0.99 (0.99–0.99)	<0.001 *	0.99 (0.99–1.00)	0.03 *
Management Type				
Surgery Receipt	1 ^#^		1 ^#^	
Surgery Refusal	3.88 (3.17–4.75)	<0.001 *	2.28 (1.84–2.83)	<0.001 *

HR = Hazard ratio. 95% CI = 95% confidence interval. C/D Index = Charlson-Deyo Comorbidity Index. * Significant *p*-value. ^#^ 1 is the reference value.

## Data Availability

Not applicable.
